# Supplementary material for: Are cell wall traits a component of the succulent syndrome?
Source: Front Plant Sci. 2022 Nov 25;13:1043429. doi: 10.3389/fpls.2022.1043429 (PMC9732111; doi:10.3389/fpls.2022.1043429)

**Supplementary figures**

**FIGURE S1.** Scatterplot matrix of the first three dimensions of MFA performed on CoMPP results from 10 succulent (S) and 10 non-succulent (NS) species. These two groups differ significantly in dimension 3 (Wilcoxon test, *p* < 0.01). On the right, contribution plots of the CoMPP fractions (water, CDTA and NaOH) to each of the MFA dimensions.

**FIGURE S2.** Scatterplot matrix of the first three dimensions of MFA omitting the three succulent taxa belonging to the Portulacineae (*Anacampseros namaquensis*, *Lithops karasmontana* and *Portulacaria afra*). Succulents and non-succulents still differ significantly in dimension 1 (Wilcoxon test, *p* < 0.01).

**FIGURE S3.** Variable importance plots from the Random Forest algorithm based on **(A)** the mean decrease of accuracy and **(B)** the mean decrease of the Gini impurity metric, showing the 30 most important variables (out of 98 CoMPP variables which yielded signal).

Figure S1


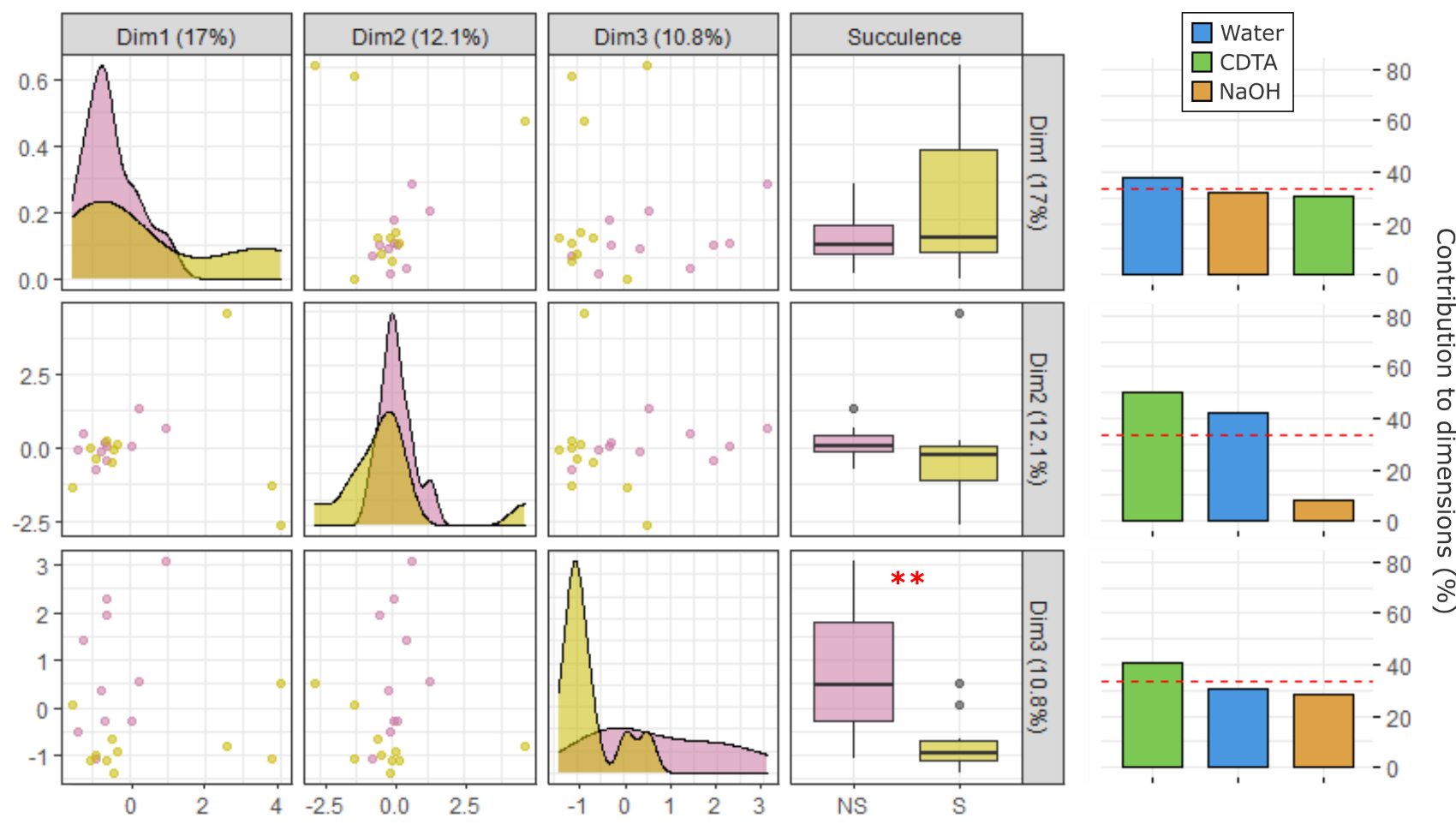


Figure S2


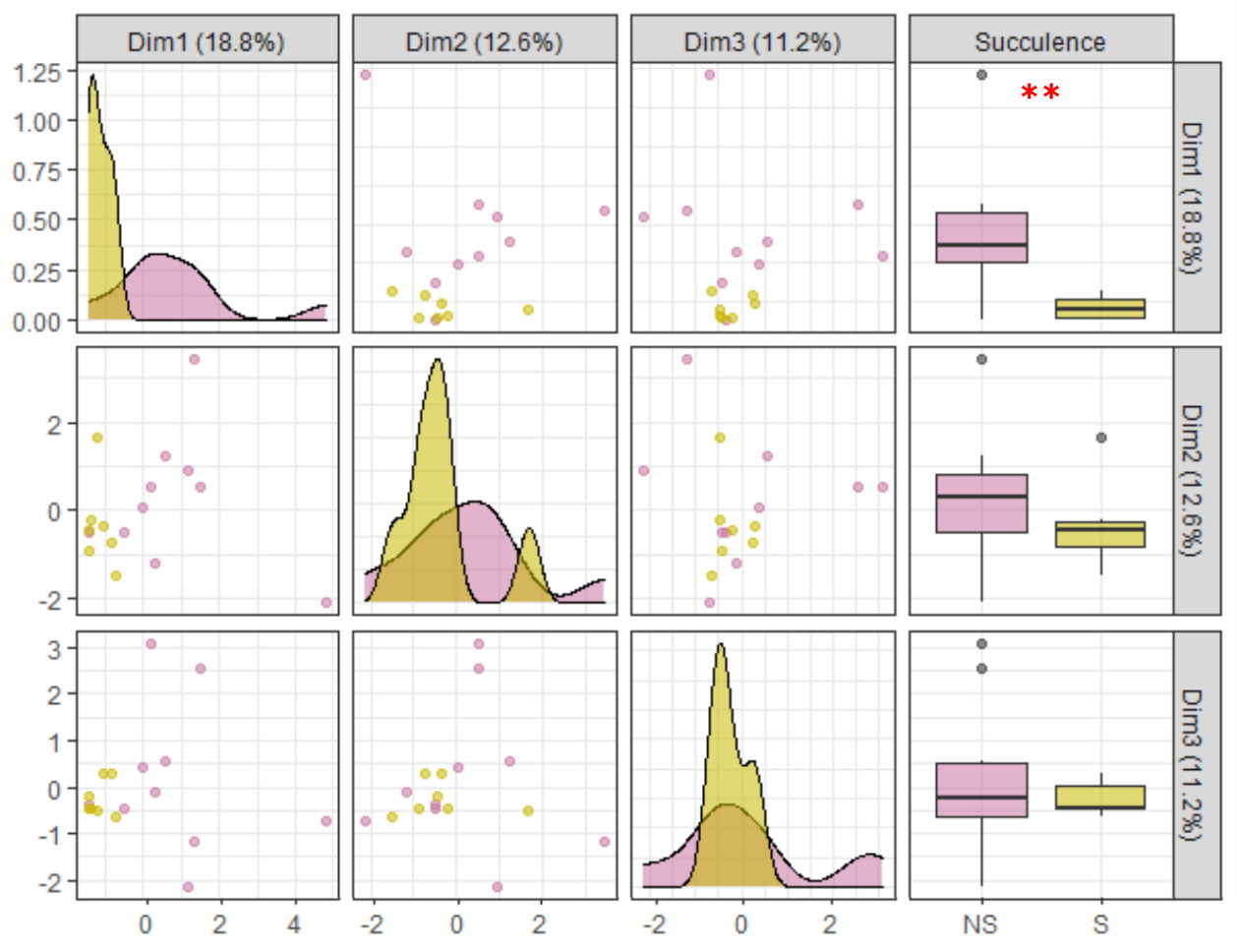


Figure S3


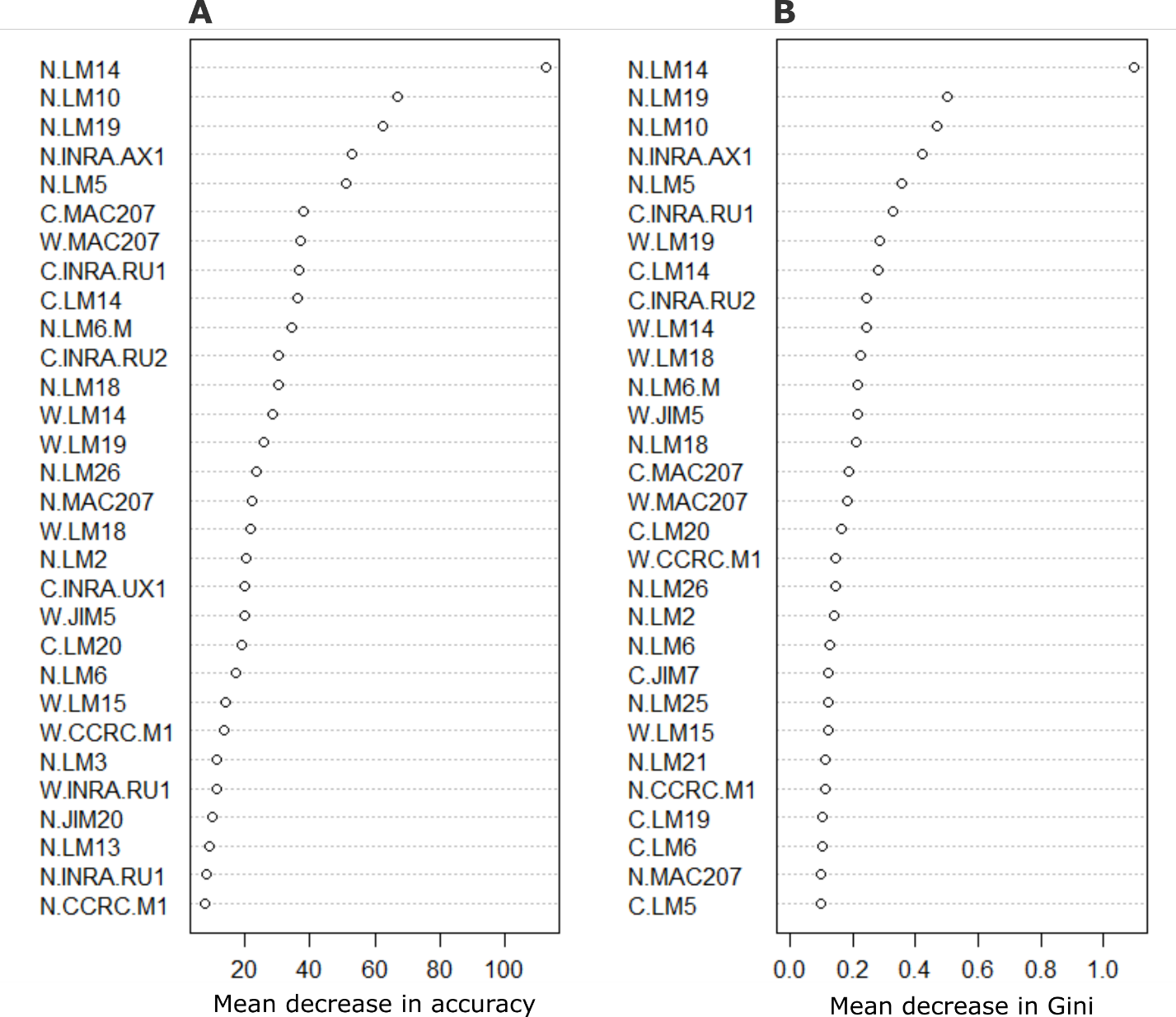

Supplement: Supplementary file 1 [file DataSheet_1.zip › Supplementary Figures.DOCX]
