# Supplementary material for: Are cell wall traits a component of the succulent syndrome?
Source: Front Plant Sci. 2022 Nov 25;13:1043429. doi: 10.3389/fpls.2022.1043429 (PMC9732111; doi:10.3389/fpls.2022.1043429)
Supplement: Supplementary file 1 [file DataSheet_1.zip › Supplementary Tables.DOCX]

**Supplementary tables**

**TABLE S1.** Angiosperm species used in this study. Abbreviations for the source of plant material: Department of Plant and Environmental Sciences, University of Copenhagen (PLEN), Kakteen-Haage nursery (KH).

| **Plant species** | **Abbreviation** | **Family** | **Angiosperm clade** | **Succulent leaves?** | **Source of material** |
| --- | --- | --- | --- | --- | --- |
| *Arabidopsis thaliana* | A_tha | Brassicaceae | Eudicot | No | PLEN |
| *Chenopodium quinoa* | C_qui | Amaranthaceae | Eudicot | No | PLEN |
| *Coleus scutellarioides* | C_scu | Lamiaceae | Eudicot | No | PLEN |
| *Dioscorea oppositifolia* | D_opp | Dioscoreaceae | Monocot | No | PLEN |
| *Epipremnum aureum* | E_aur | Araceae | Monocot | No | PLEN |
| *Jasminum mesnyi* | J_mes | Oleaceae | Eudicot | No | PLEN |
| *Leucanthemum maximum* | L_max | Asteraceae | Eudicot | No | PLEN |
| *Nicotiana benthamiana* | N_ben | Solanaceae | Eudicot | No | PLEN |
| *Viola hederacea* | V_hed | Violaceae | Eudicot | No | PLEN |
| *Vitis vinifera* | V_vin | Vitaceae | Eudicot | No | PLEN |
| *Aloe distans* | A_dis | Asphodelaceae | Monocot | Yes | PLEN |
| *Anacampseros namaquensis* | A_nam | Anacampserotaceae | Eudicot | Yes | KH |
| *Crassula ovata* | C_ova | Crassulaceae | Eudicot | Yes | PLEN |
| *Hoya australis* | H_aus | Apocynaceae | Eudicot | Yes | PLEN |
| *Kalanchoe millotii* | K_mil | Crassulaceae | Eudicot | Yes | PLEN |
| *Lithops karasmontana* | L_kar | Aizoaceae | Eudicot | Yes | KH |
| *Nematanthus gregarius* | N_gre | Gesneriaceae | Eudicot | Yes | PLEN |
| *Peperomia asperula* | P_asp | Piperaceae | Magnoliid | Yes | KH |
| *Portulacaria afra* | P_afr | Didiereaceae | Eudicot | Yes | PLEN |
| *Senecio crassissimus* | S_cra | Asteraceae | Eudicot | Yes | PLEN |

**TABLE S2.** Values of succulence index (SI) for the species used in this study. Colour intensity reflects SI values.

|  | **Species** | **Succulence index (SI)** |
| --- | --- | --- |
| **Non-succulents** | *Arabidopsis thaliana* | 10.41 |
|  | *Chenopodium quinoa* | 7.83 |
|  | *Coleus scutellarioides* | 13.51 |
|  | *Dioscorea oppositifolia* | 7.63 |
|  | *Epipremnum aureum* | 12.83 |
|  | *Jasminum mesnyi* | 2.12 |
|  | *Leucanthemum maximum* | 6.58 |
|  | *Nicotiana benthamiana* | 6.27 |
|  | *Viola hederacea* | 7.96 |
|  | *Vitis vinifera* | 1.84 |
| **Succulents** | *Aloe distans* | 27.41 |
|  | *Anacampseros namaquensis* | 15.42 |
|  | *Crassula ovata* | 12.15 |
|  | *Hoya australis* | 9.16 |
|  | *Kalanchoe millotii* | 15.34 |
|  | *Lithops karasmontana* | 11.05 |
|  | *Nemathantus gregarius* | 12.38 |
|  | *Peperomia asperula* | 30.80 |
|  | *Portulacaria afra* | 22.98 |
|  | *Senecio crassissimus* | 20.32 |

**TABLE S3.** List of primary mAbs targeting pectins. Adapted from Rydahl *et al.* (2018) and references therein, unless otherwise indicated.

| **mAb** | **Antigen / Epitope** | **Source** | **References** |
| --- | --- | --- | --- |
| **JIM5** | HG, low DM, partially methyl-esterified or de-esterified | Rat |  |
| **JIM7** | HG, high DM | Rat |  |
| **LM7** | HG, partially methyl-esterified (non-blockwise de-esterification) | Rat |  |
| **LM18** | HG, low DM, partially methyl-esterified or de-esterified | Rat |  |
| **LM19** | HG, low DM, preferably de-esterified (more selective than JIM5) | Rat |  |
| **LM20** | HG, high DM (more selective than JIM7) | Rat |  |
| **LM8** | Xylogalacturonan | Rat |  |
| **CCRC-M13** | RG-I | Mouse | Pattathil *et al.*, 2010 |
| **INRA-RU1** | RG-I backbone (min. 6 disaccharide repeats) | Mouse |  |
| **INRA-RU2** | RG-I backbone (min. 2 disaccharide repeats) | Mouse |  |
| **LM5** | (1→4)-β-D-galactan (min. 3 galactose units at non-reducing end) | Rat |  |
| **LM6** | (1→5)-α-L-arabinan / AGP epitopes | Rat |  |
| **LM6-M (BR12)** | (1→5)-α-L-arabinan (higher affinity than LM6) | Rat | Cornuault *et al.*, 2017 |
| **LM13** | Specific subset of unbranched (1→5)-α-L-arabinan (arabinanase sensitive) | Rat |  |
| **LM16** | Processed (1→5)-α-L-arabinan (galactosidase sensitive) | Rat |  |
| **LM26** | (1→4)-β-D-galactan substituted with (1→6)-β-D-galactosyl (branched galactan) | Rat |  |

**TABLE S4.** List of primary mAbs targeting hemicelluloses. Adapted from Rydahl *et al.* (2018) and references therein, unless otherwise indicated.

| **mAb** | **Antigen / Epitope** | **Source** | **References** |
| --- | --- | --- | --- |
| **BS-400-2** | (1→3)-β-D-glucan [Callose and laminarin] | Mouse |  |
| **BS-400-3** | (1→3),(1→4)-β-D-glucan [Mixed-linkage glucan, MLG] | Mouse |  |
| **CCRC-M1** | α-L-fucosylated xyloglucan / RG-I | Mouse |  |
| **CCRC-M39** | α-L-fucosylated xyloglucan / RG-I | Mouse | Pattathil *et al.*, 2010 |
| **CCRC-M58** | Xyloglucan (XLLG motif) | Mouse | Pattathil *et al.*, 2010 |
| **LM15** | Xyloglucan (XXXG motif), non-fucosylated | Rat |  |
| **LM24** | Xyloglucan (XLLG motif) | Rat |  |
| **LM25** | Xyloglucan (XLLG, XXLG, XXXG motifs) | Rat |  |
| **INRA-AX1** | Backbone of xylans | Mouse |  |
| **INRA-UX1** | Alkali-treated glucuronoxylan, GlcA (or its 4-*O*-methyl ether) substituents | Mouse | Koutaniemi *et al.*, 2012 |
| **LM10** | (1→4)-β-D-xylan | Rat |  |
| **LM23** | Non-acetylated xylosyl residues, pectic xylogalacturonan and xylan | Rat |  |
| **LM27** | Grass glucuronoarabinoxylan (GAX) | Rat |  |
| **BS-400-4** | (1→4)-β-D-(galacto)mannan | Mouse |  |
| **CCRC-M170** | Acetylated glucomannan | Mouse |  |
| **LM21** | (1→4)-β-D-(galacto)(gluco)mannan; DP2 to DP5 | Rat |  |
| **LM22** | (1→4)-β-D-(gluco)mannan; DP2 to DP5 | Rat |  |

**TABLE S5.** List of primary mAbs targeting glycoproteins and cell wall phenolics. Adapted from Rydahl *et al.* (2018) and references therein, unless otherwise indicated.

| **mAb** | **Antigen / Epitope** | **Source** | **References** |
| --- | --- | --- | --- |
| **JIM11** | Extensin (periodate sensitive) | Rat |  |
| **JIM12** | Extensin (proteinase sensitive) | Rat |  |
| **JIM19** | Extensin (periodate sensitive) | Rat |  |
| **JIM20** | Extensin (periodate sensitive) | Rat |  |
| **LM1** | Extensin / Hydroxyproline-rich glycoproteins (HRGPs) | Rat |  |
| **LM3** | Extensin | Rat | Feng *et al.*, 2014 |
| **JIM4** | AGP (β-D-GlcA-(1→3)-α-D-GalA-(1→2)-α-D-Rha competes for binding) | Rat |  |
| **JIM8** | AGP, carbohydrate portion | Rat | Pennell *et al.*, 1991 |
| **JIM13** | AGP (β-D-GlcA-(1→3)-α-D-GalA-(1→2)-α-D-Rha competes for binding) | Rat |  |
| **JIM16** | AGP, (1→3)-β-D-galactan chain with single (1→6)-β-D-linked Gal residue | Rat |  |
| **LM2** | AGP, (1→6)-β-D-galactan chain with terminally attached GlcA | Rat |  |
| **LM14** | AGP / Arabinogalactan | Rat |  |
| **LM30** | AGP (arabinofuranosidase sensitive) | Rat | Wilkinson *et al.*, 2017 |
| **MAC207** | AGP (β-D-GlcA-(1→3)-α-D-GalA-(1→2)-α-D-Rha competes for binding) | Rat |  |
| **LM9** | Feruloylated (1→4)-β-D-galactan | Rat |  |
| **LM12** | Feruloylate/ferulic acid on any polymer and heteroxylan | Rat |  |

**References**

Cornuault, V., Buffetto, F., Marcus, S.E., Crépeau, M.-J., Guillon, F., Ralet, M.-C., and Knox, P. (2017). LM6-M: a high avidity rat monoclonal antibody to pectic α-1,5-L-arabinan. *bioRxiv* [Preprint]. doi: 10.1101/161604

Feng, T., Nyffenegger, C., Højrup, P., Vidal-Melgosa, S., Yan, K.P., Fangel, J.U., et al. (2014). Characterization of an extensin-modifying metalloprotease: N-terminal processing and substrate cleavage pattern of *Pectobacterium carotovorum* Prt1. *Appl. Microbiol. Biotechnol.* 98, 10077–10089. doi: 10.1007/s00253-014-5877-2

Koutaniemi S., Guillon F., Tranquet O., Bouchet B., Tuomainen P., Virkki L., et al. (2012). Substituent-specific antibody against glucuronoxylan reveals close association of glucuronic acid and acetyl substituents and distinct labeling patterns in tree species. *Planta* 236, 739–751. doi: 10.1007/s00425-012-1653-7

Pattathil, S., Avci, U., Baldwin, D., Swennes, A.G., McGill, J.A., Popper, Z., et al. (2010). A comprehensive toolkit of plant cell wall glycan-directed monoclonal antibodies. *Plant Physiol.* 153, 514–525. doi: 10.1104/pp.109.151985

Pennell, R.I., Janniche, L., Kjellbom, P., Scofield, G.N., Peart, J.M., Roberts, K. (1991). Developmental regulation of a plasma membrane arabinogalactan protein epitope in oilseed rape flowers. *Plant Cell* 3, 1317–1326. doi: 10.1105/tpc.3.12.1317

Rydahl, M.G., Hansen, A.R., Kračun, S.K., and Mravec, J. (2018). Report on the current inventory of the toolbox for plant cell wall analysis: proteinaceous and small molecular probes. *Front. Plant Sci.* 9:581. doi: 10.3389/fpls.2018.00581

Wilkinson, M.D., Tosi, P., Lovegrove, A., Corol, D.I., Ward, J.L., Palmer, R., et al. (2017). The *Gsp-1* genes encode the wheat arabinogalactan peptide. *J. Cereal Sci.* 74, 155–164. doi: 10.1016/j.jcs.2017.02.006
